# Dual-Electrodes PMUTs on Glasses for Wearable Human Blink Monitoring

**DOI:** 10.3390/mi17010090

**Published:** 2026-01-09

**Authors:** Xiao-Xin Liang, Haochen Wu, Yong Wang

**Affiliations:** 1 Department of Ophthalmology, The Fourth Affiliated Hospital of School of Medicine, Zhejiang University, Yiwu 322000, China; 8016046@zju.edu.cn; 2 Department of Mechanical Engineering, School of Engineering, Hangzhou City University, Hangzhou 310015, China

**Keywords:** piezoelectric film, ultrasonic, acoustic MEMS, blink monitoring, wearable sensor

## Abstract

Blink monitoring has demonstrated significant application value in fields such as safety assessments, medical monitoring, and intelligent technologies. Traditional eye monitoring methods are limited by restricted adaptability, insufficient comfort, or potential risks. MEMS-based ultrasonic technology, as a non-contact approach, has garnered attention due to its strong environmental adaptability, privacy, and security. However, existing designs require high-sensitivity processing circuits and are incompatible with standard fabrication processes. This work proposes a dual-electrode piezoelectric micro-mechanical ultrasonic transducer (PMUT) design based on aluminum nitride (AlN) piezoelectric thin films, integrated into a glasses device to enable real-time blink activity monitoring. The design successfully identifies blink states through time-of-flight (TOF) pulse-echo technology and dynamic unsupervised learning methods. Fabricated using cost-effective standard multi-user MEMS processes, this device offers distinct merits in terms of wearability comfort, information security, biosafety, and reliability.

## 1. Introduction

Blinking, as one of the most fundamental and universal physiological behaviors in humans, occurs approximately more than 15 times per minute [1]. This behavior can be categorized into two types: involuntary blinking and voluntary blinking [2]. Involuntary blinking is significantly influenced by psychological and physiological states, while voluntary blinking is highly controllable [3]. Its importance extends beyond basic physiological needs, offering unique application value in human interaction, medical monitoring, and intelligent technologies. In the field of traffic safety, the frequency and duration of involuntary blinking hold practical significance. Drowsy driving is one of the primary causes of traffic accidents, and real-time monitoring of a driver’s blinking activity, particularly the prolongation of closed-eye time and an increase in blinking frequency, can effectively assess the driver’s state of fatigue, thereby reducing accident rates and ensuring traffic safety [4]. In the medical field, involuntary blinking behavior also serves as an important assessment tool. For example, in the rehabilitation of Bell’s palsy and facial nerve injury patients, analyzing the patient’s blinking frequency, closed-eye duration, and blink completeness provides an accurate means of evaluating nerve repair and recovery progress. This non-invasive assessment method offers significant benefits for clinical diagnosis and treatment outcomes [5]. In the realm of technology, voluntary blinking demonstrates unique interaction value. For instance, in special combat or covert operations, soldiers can communicate through specific blinking patterns without language or gestures, enabling the silent transmission of critical information without affecting their actions [6]. Additionally, in medical settings, voluntary blinking provides an alternative communication method for patients unable to use language or gestures. When combined with computer systems, blinking actions can be captured and converted into specific input commands, offering disabled individuals a new means of controlling devices and improving their quality of life [7]. In the consumer electronics sector, the integration of blink monitoring technology with eye-tracking systems provides technical support for immersive interaction in augmented reality (AR) headsets and smart glasses, laying the foundation for innovative interaction methods in future intelligent devices [8].

However, commonly used eye movement monitoring methods such as Video Oculography (VOG), Electrooculography (EOG), and Infrared Oculography (IROG) each have limitations. While VOG has strong environmental adaptability, its reliance on cameras can result in poor performance under extreme lighting conditions, and long-term face monitoring may raise privacy concerns [9]. EOG relies on sensors attached to the skin surface for monitoring, offering strong adaptability, but prolonged use may cause discomfort [10]. In comparison, IROG avoids direct skin contact, eliminating comfort-related issues, but prolonged exposure to infrared light may lead to eye dryness, fatigue, and even potential safety risks [11].

Ultrasonic technology, as an emerging monitoring method, has garnered significant attention due to its unique advantages [12,13,14,15]. It has been widely used in medical imaging, industrial inspection, and ranging, and has demonstrated strong adaptability under extreme environmental and complex lighting conditions [16,17,18]. Importantly, when used solely for ranging, ultrasonic technology does not collect facial information, effectively protecting personal privacy. Furthermore, ultrasonic technology has been successfully applied in eye imaging techniques, with low-intensity ultrasound proven not to cause damage to the eyes, significantly enhancing its application safety [19,20]. These advantages make ultrasonic technology a highly promising alternative, particularly suitable for long-term monitoring scenarios. However, traditional ultrasonic transducers, due to their large size and significant weight, struggle to meet the requirements of portable applications [21]. Against this backdrop, with the rapid advancement of Micro-Electro-Mechanical Systems (MEMS) technology, compact and lightweight MEMS ultrasonic transducers have become a focal point of research [22,23,24]. Their millimeter-level size attributes facilitate their integration into eyeglass lenses or frames, offering a novel solution for human blink monitoring [25]. The classic design of piezoelectric micro-mechanical ultrasonic transducers (PMUTs) for blink monitoring has evolved from the single-electrode architecture, which enables operation in both TX (transmission) and RX (reception) modes through mode switching [26], while the emergence of dual-electrode PMUTs has demonstrated theoretically promising performance for short-range detection applications [27,28], with dedicated electrodes for signal excitation and echo reception. However, there are no literature reports on the feasibility and applicability of their integration with wearable devices.

In this work, we propose a dual-electrode PMUT design based on aluminum nitride (AlN) piezoelectric thin films, achieving independent isolation of TX and RX functions within a single PMUT. This design overcomes the limitations of TX-RX mode switching in conventional single-electrode PMUTs and significantly enhances the system’s stability and reliability. Leveraging the excellent CMOS compatibility and ease of microfabrication offered by AlN, this design demonstrates a notable cost-effectiveness advantage. Additionally, the PMUT device measures only hundreds of micrometers in size, enabling seamless integration into wearable eyewear without interfering with the user’s normal vision. Through the implementation of time of flight (ToF) pulse-echo technology and dynamic unsupervised learning methods, this study successfully achieves intelligent identification of blink states, providing an efficient solution for portable human blink monitoring. Compared to traditional methods, our proposed precept offers significant merits in terms of wearability comfort, information security, biological safety, and reliability. These advancements lay a solid foundation for the widespread application of this technology in fields such as medical monitoring, human–computer interaction, and intelligent devices in the future.

## 2. Results and Discussion

### 2.1. Conceptualization of PMUT-Based Blink Monitoring

**Figure 1:**
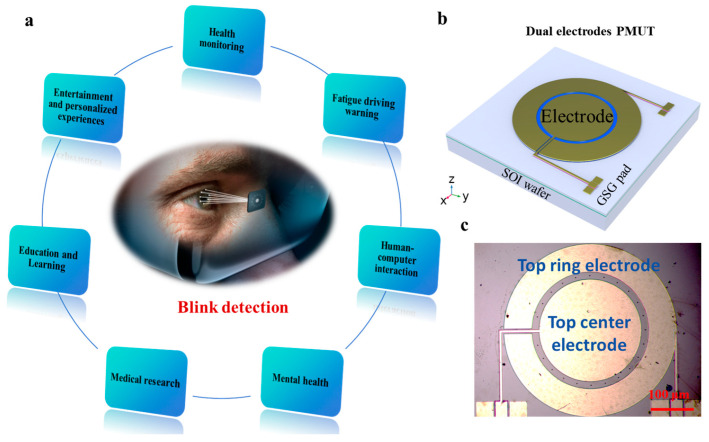
(**a**) Conceptual diagram of blink detection using MEMS ultrasonic chips and application examples. (**b**) 3D design schematic diagram of a dual-electrode PMUT. (**c**) Microscopic optical image of fabricated dual-electrode PMUTs.

As shown in Figure 1a, blink monitoring technology demonstrates vast potential across multiple domains [29]. In health monitoring, real-time tracking of blink activity enables effective identification of fatigue states, providing technical support to address the issue of drowsy driving. In the realm of mental health, abnormal changes in blinking behavior can serve as a critical reference for assessing an individual’s psychological state. In medical research, analyzing blink frequency and closed-eye duration offers a scientific basis for evaluating the rehabilitation progress of patients with nerve injuries. Furthermore, this technology holds significant value in human–computer interaction, educational learning, entertainment, and personalized experiences. However, current blink monitoring methods still face challenges in achieving high levels of portability and wearability. Traditional monitoring devices are often large in size and unsuitable for prolonged use, limiting their widespread application in daily scenarios [30]. Thus, developing a solution that ensures monitoring precision while maintaining high portability and wearability is of utmost importance.

Ultrasound technology, as a highly biocompatible and non-invasive detection method, provides a new approach to addressing this challenge. Its non-intrusive nature, high sensitivity, and independence from environmental light conditions make it particularly advantageous for blink monitoring. Notably, low-intensity ultrasound has been proven safe and harmless to ocular tissues, further enhancing its applicability. Piezoelectric thin film-based silicon resonators serve as an ideal platform for implementing this technology, offering several key advantages: their compact size and lightweight design enable seamless integration into eyeglass lenses or frames, with negligible impact on the wearer; their simple and reliable operating principle provides a robust foundation for stable and real-time monitoring [31].

### 2.2. Design and Fabrication of Dual-Electrode PMUTs

**Figure 2:**
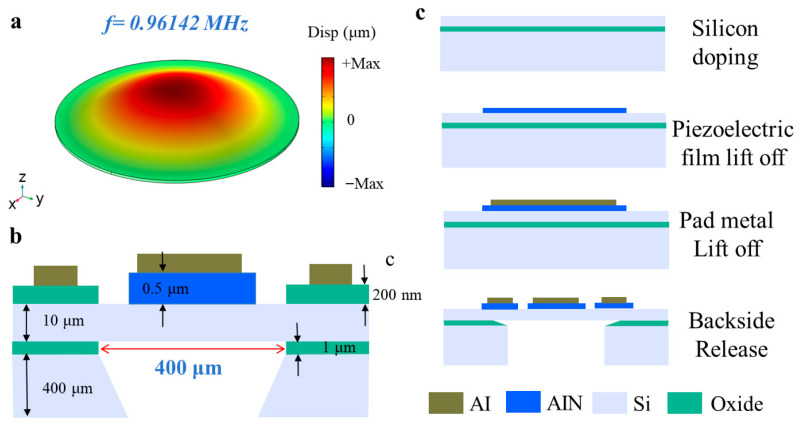
(**a**) (0, 0) mode deformation profile and resonance frequency of the PMUT device. (**b**) Cross-sectional multilayer structure diagram and parameter dimensions of the (0, 0) mode PMUT device. (**c**) Standard MEMS manufacture flowchart for PiezoMEMS devices.

As shown in Figure 1b,c, the 3D design schematic and fabricated microscope optical image of the dual-electrode PMUT illustrate its structural details. The PMUT employs aluminum nitride (AlN) as the piezoelectric film, enabling the creation and recognition of ultrasound waves. Compared to commonly used lead zirconate titanate (PZT) materials, AlN exhibits distinct advantages: it has higher receive sensitivity, easier integration with complementary metal-oxide-semiconductor (CMOS) circuits, and is non-toxic and free of lead, making it environmentally friendly and particularly well-suited for portable applications [32]. The PMUT features a circular diaphragm design, activated in the fundamental bending mode, as shown in Figure 2a. The maximum strain regions are located at the center and clamped edges of the diaphragm. The strain amplitudes between the center and clamped edges are similar but with opposite phases. Each electrode covers one of these maximally strained and phase-opposite regions. In the dual-electrode configuration, one electrode is positioned at the diaphragm’s center, while the other surrounds the edge of the diaphragm. Both electrodes share a common bottom ground electrode formed by a highly doped silicon layer. The diaphragm thickness is set to 10 μm, offering a balanced compromise between coupling efficiency and diaphragm size. Thicker diaphragms provide larger emission areas and higher output pressures at the same resonance frequency but result in lower coupling efficiency. According to finite element (FE) simulations using COMSOL Multiphysics 6.1, the circular PMUT (with a diameter of 400 μm) achieves a resonance frequency of 961.42 kHz in its fundamental mode. When an alternating voltage at this specific frequency is applied across the pair of electrodes, the piezoelectric material induces tensile stress as a result of the inverse piezoelectric effect, thereby exciting the bending oscillation of the structural diaphragm.

As shown in Figure 2b,c, this device is fabricated using the PiezoMUMPs process provided by MEMSCAP [33]. The 400 μm diameter PMUT circular membrane is released from the backside of the silicon-on-insulator (SOI) wafer using deep reactive ion etching (DRIE) to create a cavity. The SOI wafer consists of a 400 μm thick handle layer, a 1 μm thick buried oxide layer, and a 10 μm thick silicon device layer. Within the circular membrane, a 0.5 μm thick AlN piezoelectric layer and a 20/1000 nm thick chromium/aluminum (Cr/Al) metal layer are deposited, followed by patterning on the AlN layer. The two electrodes are structured as a central circular electrode and an annular electrode. A doped Si device layer is used as the bottom ground electrode for Ohmic contact. A 200 nm thick thermal oxide layer is deposited and patterned to insulate the contact pads from the silicon device layer.

### 2.3. PMUT Blink Monitoring Principle

**Figure 3:**
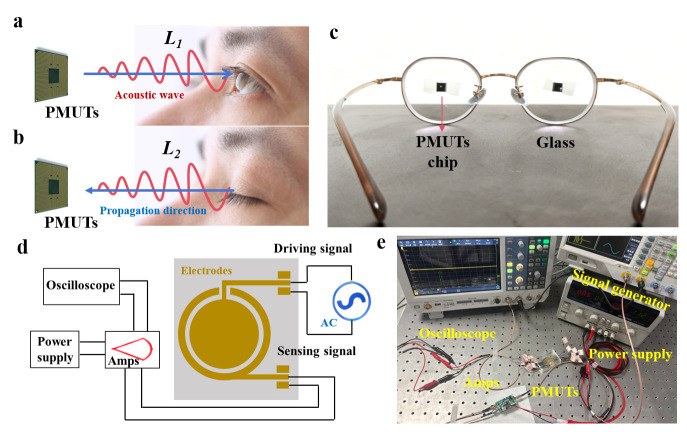
(**a**,**b**) Schematic illustration of the propagation of an ultrasonic wave when the eyes are open or closed. (**c**) Integration of two PMUT array chips mounted on a pair of glasses. (**d**) Schematic diagram of the test circuit for dual-electrode PMUTs. (**e**) A physical photo of the actual test system.

The dual-electrode PMUT functions by being electrically actuated in air, prompting it to vibrate and generate ultrasonic waves through the inverse piezoelectric effect. These ultrasonic waves, upon encountering the eyes or eyelids, are primarily reflected and travel back to the dual-electrode PMUT along their original path, as illustrated in Equation (1) [26]. This phenomenon occurs because the acoustic impedance of the human eye is notably greater than that of air. Upon receiving the ultrasonic vibrations, the dual-electrode PMUT captures the received signals via the piezoelectric effect. As shown in Figure 3a, when the eyes are open, the ultrasonic waves are reflected by the eye surface after a propagation distance of *L*_1_. Conversely, when the eyes are closed, as depicted in Figure 3b, the ultrasonic waves are reflected upon interacting with the eyelid, resulting in a propagation distance of *L*_2_. The eye-opening and closing states correspond to different propagation times due to the eyelid’s variable thickness. By evaluating the flight times of the pulse-echo signals, we can effectively discriminate between open and closed eye conditions. The time difference between these states is expressed in Equation (2) [26]:

(1) $$T=\frac{{2(Z}_{2}-Z_{1})}{Z_{2}+Z_{1}}$$

(2) $$t=\frac{2(L_{1}-L_{2})}{c}$$

where T represents the reflection coefficient, Z_1_ is the acoustic impedance of the eyelid or eye, and Z_2_ is the acoustic impedance of air in the environment.

### 2.4. PMUT Blink Monitoring Circuit Test System

The experimental setup, as depicted in Figure 3c, comprises mounting two miniaturized chips fabricated with nine dual-electrode PMUTs array on a pair of glasses (e.g., only one PMUT unit was used for experiment), with the device scaled down to suit practical applications. The typical separation between the glasses and the eyes is approximately 9 to 12 mm. The schematic principles and physical diagrams of the test circuit are illustrated in Figure 3d. This circuit is composed of two primary components: the transmit (TX) driver section and the receive (RX) section. The TX driver section employs a programmable signal generator to produce the requisite pulse wave signals. In the RX section, the pulse-echo signals are amplified by a differential amplifier (AD8331) and subsequently captured by a commercial oscilloscope (RTB 2004, Rohde & Schwarz, Munich, Germany) for further processing and digitization. The AD8331 (Analog Devices, Wilmington, DE, USA) is a single-channel, ultra-low-noise amplifier specifically designed for ultrasonic systems. This amplifier incorporates an ultra-low-noise low-noise amplifier (LNA), a variable gain amplifier (VGA) with a gain range of 48 dB, and a selectable gain post-amplifier that includes adjustable output limiting functionality. A power supply was used to power the amplifier. The physical photos of the actual test system are recorded in Figure 3e.

### 2.5. Electrical Characterization of Dual-Electrodes PMUT

The resonant frequency of the PMUT device is governed by the radius r of backside cavity and structural thickness t, adhering to the following frequency formula:

(3) $$F\propto \sqrt{\frac{k_{m}}{m_{m}}}\propto \frac{t}{r^{2}}$$

where km denotes the equivalent stiffness and mm represents the equivalent mass of the first-order mode.

**Figure 4:**
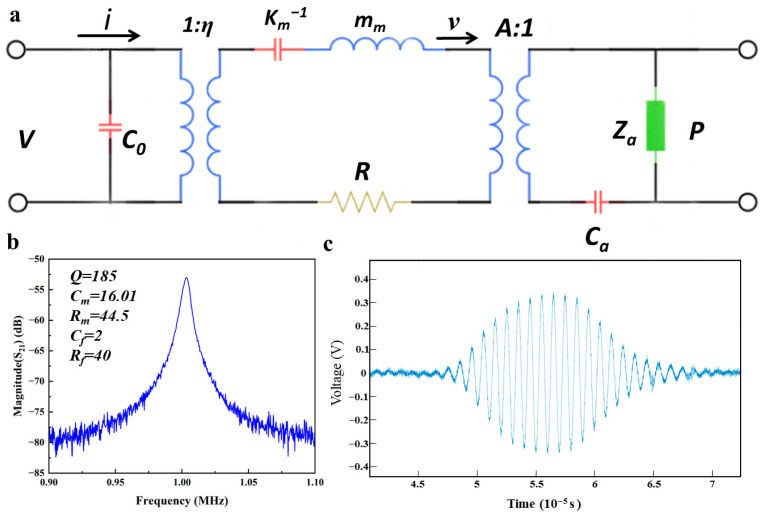
(**a**) Equivalent circuit model of a single PMUT device. (**b**) Frequency response S21 curve of PMUT devices and characterization of performance parameters. (**c**) Amplified acoustic echo signal of PMUT in time domain when applying the input with 10 cycles and 12 Vpp.

To facilitate analytical investigations, an equivalent circuit model was developed, as illustrated in Figure 4a. The components of this model are defined as follows:

• *C*_0_ signifies the static capacitance of the PMUR device,
• *R* represents the mechanical damping inherent within the device,
• *A* denotes the effective surface area of the structural membrane,
• *C* stands for the acoustical compliance of the cavity,
• *η* is the electromechanical coupling coefficient,
• *Z* is the acoustic impedance.

When the circular plate operates in the fundamental (0, 0) vibration mode, the equivalent parameters of the mechanical subsystem are mathematically expressed as follows [34]:

(4) $$k_{m}=\frac{64\pi D}{3r^{2}}$$

(5) $$m_{m}=\frac{\pi r^{2}\mu }{5}$$

(6) $$\eta =4\pi \gamma^{2}(\gamma^{2}-1)e_{31,f}{\bar{Ζ}}_{p}$$

where D is the bending stiffness of the structure, μ represents the mass per unit area, r is the cavity radius, and γ is the ratio of the top electrode radius to the cavity radius.

The frequency response of the dual-electrode PMUT was measured using a network analyzer, with the results illustrated in Figure 4b. The experimental data indicate that the resonant frequency of the PMUT device is 1003 kHz, which is a compromise choice because higher frequencies will intensify the attenuation of ultrasonic waves in the air, while lower frequencies will reduce the temporal resolution. Note that the over etching (e.g., ≤30 µm) of DRIE process of the PMUT back cavity bring processing errors between the designed dimensions, affecting the film diameter and slightly altering the resonant frequency. The primary source of error arises from the deviation in the verticality during the back-cavity etching process, which resulted in the film’s actual diameter being marginally larger than the designed specifications. Additionally, comprehensive analyses of resonance parameters were conducted, encompassing the quality factor, dynamic resistance, dynamic capacitance, parasitic resistance, and parasitic capacitance.

Figure 4c illustrates the amplified time-domain acoustic echo signal of the dual-electrode PMUT when subjected to 10 cycles with an input voltage of 12 Vpp. This signal demonstrates that the device achieves a complete vibration state, corroborating its effective operational capabilities.

### 2.6. PMUT Blink Test Under Eyelid Opening and Closing States

#### 2.6.1. Single-Eye Opening/Closing Test

The single-eyelid opening and closing states were detected using an ultrasonic pulse-echo system. The system configuration is as follows: the input Excitation signal has an amplitude of 12 Vpp, 10 cycles, with a pulse repetition interval (PRI) of 10 ms. The ultrasonic frequency is set to 1003 kHz, corresponding to a wavelength of 339 μm, and the system’s longitudinal resolution is 848 μm.

**Figure 5:**
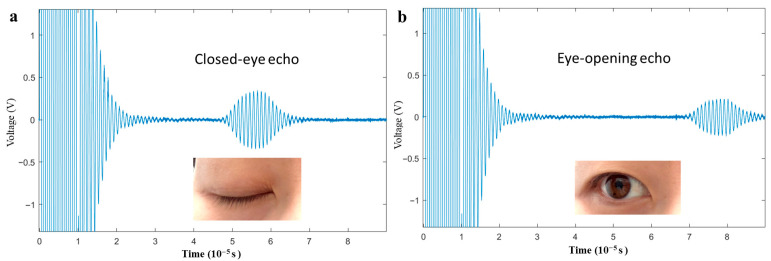
(**a**,**b**) The pulse echo curves of dual-electrodes PMUT when one eye is open or closed.

As shown in Figure 5, the pulse-echo signal characteristics differ distinctly between the eyes opening and closing states. In the open state (Figure 5b), the Time of Flight (TOF) is 79 μs, the start time is 72 μs, and the receive voltage Vpp is 180 mV. In the closed state (Figure 5a), the TOF is 56 μs, the start time is 50 μs, and the receive voltage Vpp is 270 mV. Based on the speed of sound, the displacements of the eyelid in the open and closed states are calculated to be 8.50 mm and 10.54 mm, respectively. It is important to note that while TOF is used to characterize the echo time features, the TOF specifically refers to the time corresponding to the peak of the echo signal in this context.

#### 2.6.2. Synchronized Blinks in Both Eyes

**Figure 6:**
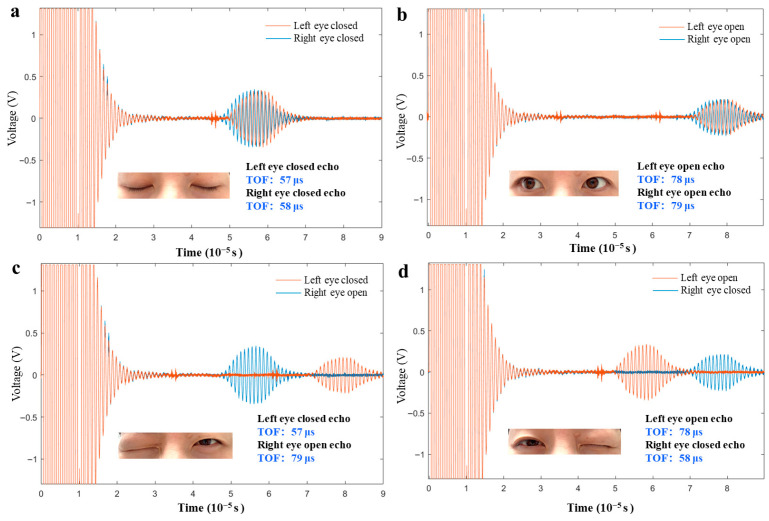
(**a**,**b**) The pulse echo curves of dual-electrodes PMUT when two eyes are open or closed. (**c**,**d**) The pulse echo curves of dual-electrodes PMUT when left eye closed and the right eye opened or right eye closed and the left eye opened.

The synchronized opening and closing states of both eyes were tested, with results shown in Figure 6a,b. When both eyes are open, the TOF values are 78 μs for the left eye and 79 μs for the right eye. When both eyes are closed, the TOF values are 57 μs for the left eye and 58 μs for the right eye. The TOF difference between the left and right eyes in both open and closed states is consistently 21 μs, demonstrating good consistency. This minor time difference primarily arises from the slight variations in local shape caused by differences in the muscle tension of the eyelids on both sides, which has negligible effects on the overall blink monitoring.

#### 2.6.3. Single-Eyelid Closure Test

Additional experiments were conducted to validate the detection accuracy in single-eyelid closure states. The results, as illustrated in Figure 6c,d, are as follows: (1) When the left eye is closed and the right eye is open, the TOF values are 57 μs (left eye) and 79 μs (right eye), with a difference of 22 μs. (2) When the left eye is open and the right eye is closed, the TOF values are 78 μs (left eye) and 58 μs (right eye), with a difference of 20 μs. This symmetrical difference in TOF values confirms the system’s sensitivity to single-eyelid closure states. The 2 μs discrepancy is primarily attributed to the subtle changes in muscle tension of the eyelids when closed, which have minimal impact on the overall performance of the blink detection system. Therefore, the experimental results demonstrate that the ultrasonic detection system is capable of effectively distinguishing between different eyelid opening and closing states, achieving high sensitivity and accuracy.

In exploring the practicality of integrating PMUTs into wearable devices, it is essential to ensure that their implementation causes as little disruption as possible to the user’s natural field of vision and line of sight. To address this, three key considerations come into play. Firstly, embedding a single PMUT module within the lens can help maintain an unobstructed view. Secondly, transparent conductive electrodes can be employed to route the circuitry from the PMUT to the frame, thus reducing the overall lens area required for the device. The most effective packaging solution, however, involves encapsulating the PMUT within the lens using vacuum technology. This method not only enhances operational efficiency through improved resonance but also provides durable protection against environmental factors such as moisture and fogging. Together, these approaches aim to balance functionality with user experience, paving the way for practical and scalable applications in the future.

## 3. Conclusions

This work successfully demonstrates the feasibility of an innovative solution based on dual-electrode PMUTs for emitting and receiving ultrasound waves to monitor eye blinking. The device boasts significant technical advantages due to its miniaturization, light weight, and low power consumption. With a dimensional size of less than 1 mm, it can be seamlessly integrated into eyewear devices without affecting the wearer’s vision. By integrating the time-of-flight pulse-echo technique and dynamic unsupervised learning methods, the system achieves intelligent identification of blink states with high efficiency. Experimental results validate the system’s superior recognition capabilities across three static states: one eye open while the other is closed, both eyes open, and both eyes closed simultaneously. These findings conclusively demonstrate the system’s reliability and accuracy in practical applications. In terms of key performance metrics, this system exhibits notable advantages. Firstly, its ultra-miniaturized design makes it highly suitable for integration into wearable devices. Secondly, in terms of information security and biosafety, the non-invasive nature of ultrasound technology offers enhanced privacy protection. Furthermore, the system demonstrates outstanding stability and reliability, fully meeting the stringent precision requirements of blink monitoring. These advantages position the system to offer expansive application prospects in smart eyewear, medical monitoring, human–computer interaction, and beyond.

## Data Availability

The original contributions presented in this study are included in the article. Further inquiries can be directed to the corresponding author(s).

## References

[1] Matteo B., Giulia P., Josep V., Mark H., Alfredo B. (2024). Neural control of blinking. Clin. Neurophysiol..

[2] Hoppe D., Helfmann S., Rothkopf C.A. (2018). Humans quickly learn to blink strategically in response to environmental task demands. Psychol. Cogn. Sci..

[3] Huber S.E., Martini M., Sachse P. (2022). Patterns of eye blinks are modulated by auditory input in humans. Cognition.

[4] Shaik M.E. (2023). A systematic review on detection and prediction of driver drowsiness. Transp. Res. Interdiscip. Perspect..

[5] Oganov A., Yazdanpanah G., Jabbehdari S., Belamkar A., Pflugfelder S. (2023). Dry eye disease and blinking behaviors: A narrative review of methodologies for measuring blink dynamics and inducing blink response. Ocul. Surf..

[6] Descroix E., Świątkowski W., Graff C. (2022). Blinking While Speaking and Talking, Hearing, and Listening: Communication or Individual Underlying Process?. J. Nonverbal Behav..

[7] Fan L.-H., Huang W.-C., Shao X.-Q., Niu Y.-F. (2024). Design recommendations for voluntary blink interactions based on pressure sensors. Adv. Eng. Inform..

[8] Klaib A.F., Alsrehin N.O., Melhem W.Y., Bashtawi H.O., Magableh A.A. (2021). Eye tracking algorithms, techniques, tools, and applications with an emphasis on machine learning and Internet of Things technologies. Expert Syst. Appl..

[9] Lawand S.A. (2024). Eye tracking techniques and medical applications: A comprehensive review. Int. J. Sci. Res. Arch..

[10] Haslwanter T., Clarke A.H. (2010). Chapter 5-Eye movement measurement: Electro-oculography and video-oculography. Handb. Clin. Neurophysiol..

[11] Zhu L., Chen J., Yang H., Zhou X., Gao Q., Loureiro R., Gao S., Zhao H. (2024). Wearable Near-Eye Tracking Technologies for Health: A Review. Bioengineering.

[12] Wang Y., Lai X., Chen Q., Han X., Lu L., Ouyang M., Zheng Y. (2024). Progress and challenges in ultrasonic technology for state estimation and defect detection of lithium-ion batteries. Energy Storage Mater..

[13] Zhou S., Park G., Lin M., Yang X., Xu S. (2025). Wearable ultrasound technology. Nat. Rev. Bioeng..

[14] Wang M., Jia L., Li H., Feng X. (2025). Wearable Ultrasound Devices for Biomedical Applications. FlexTech.

[15] Li R., Wang F., Yi P., Yang F., Zhao J., Yue Z., Liu L., Sfarra S., Vesala G.T., Yue H. (2025). A review of ultrasonic infrared thermography in non-destructive testing and evaluation (NDT&E): Physical principles, theory, and data processing. Infrared Phys. Technol..

[16] Cao X., Yang H., Wu Z.-L., Li B.-B. (2024). Ultrasound sensing with optical microcavities. Light Sci. Appl..

[17] Xu X., Ran B., Jiang N., Xu L., Huan P., Zhang X., Li Z. (2024). A systematic review of ultrasonic techniques for defects detection in construction and building materials. Measurement.

[18] Wen H., Cheng D., Chen Y., Yue W., Zhang Z. (2024). Review on ultrasonic technology enhanced biological treatment of wastewater. Sci. Total Environ..

[19] Poinard S., Ganeau A., Lafond M., Dorado O., Catheline S., Lafon C., Aptel F., Thuret G., Gain P. (2024). Ultrasound application in Ophthalmology: A review. IRBM.

[20] Wang X., Li Z., Zhou S. (2024). Development of a High-Frequency Ophthalmic Real-Time Ultrasound Imaging System Based on a 20 MHz Annular-Array Transducer. J. Eng..

[21] Huang H., Wu R.S., Lin M., Xu S. (2024). Emerging Wearable Ultrasound Technology. IEEE Trans. Ultrason. Ferroelectr. Freq. Control.

[22] Wu C., Guan Z., Hu N., Duan X., Cui D., Wang Y. (2025). An AlN-Based Piezoelectric Ultrasonic Transducer Structure with Low Residual Stress. IEEE Sens. J..

[23] Pernu T., Sillanpää T., Baby Karuthedath C., Sebastian A.T. (2024). Development of PMUT-Based High Sensitivity Gas Flow Sensor. J. Microelectromech. Syst..

[24] Pan J., Bai C., Zheng Q., Xie H. (2023). Review of Piezoelectric Micromachined Ultrasonic Transducers for Rangefinders. Micromachines.

[25] Wang Y., Chen P., Zhang J., Yu H. (2024). Quasi-closed diaphragm based piezoelectric micromachined ultrasonic transducer with reduced Q and stress sensitivity for in-air rangefinding. Sens. Actuators A Phys..

[26] Sun S., Wang J., Zhang M., Ning Y., Ma D., Yuan Y., Niu P., Rong Z., Wang Z., Pang W. (2022). MEMS ultrasonic transducers for safe, low-power and portable eye-blinking monitoring. Microsyst. Nanoeng..

[27] Zhang Y., Miao B., Wang G., Zhou H., Zhang S., Hu Y., Wu J., Yu X., Li J. (2023). ScAlN Film-Based Piezoelectric Micromechanical Ultrasonic Transducers with Dual-Ring Structure for Distance Sensing. Micromachines.

[28] Suresh A., Mak K., Benserhir J., Lee E.-Y. Air-coupled Ultrasonic Rangefinder with Meter-long Detection Range Based on a Dual-electrode PMUT Fabricated Using a Multi-user MEMS Process. Proceedings of the 2019 IEEE SENSORS.

[29] Chen R., Zhang Z., Deng K., Wang D., Ke H., Cai L., Chang C.-W., Pan T. (2021). Blink-sensing glasses: A flexible iontronic sensing wearable for continuous blink monitoring. iScience.

[30] Yi X., Jia J., Deng S., Shen S.G., Xie Q., Wang G. (2013). A Blink Restoration System with Contralateral EMG Triggered Stimulation and Real-Time Artifact Blanking. IEEE Trans. Biomed. Circuits Syst..

[31] Yao S., Shang W., Ta G., Tao J., Liu H., Zhao X., Liu J., Miao B., Li J. (2024). ScAlN PMUTs Based on Flexurally Suspended Membrane for Long-Range Detection. Micromachines.

[32] Piazza G., Felmetsger V., Muralt P., Olsson R.H., Ruby R. (2012). Piezoelectric aluminum nitride thin films for microelectromechanical systems. MRS Bull..

[33] (2013). PiezoMUMPs Design Handbook.

[34] Reddy J.N., Soares C.A.M., Soares C.M.M., Freitas M.J.M. (1999). Theory and Analysis of Laminated Composite Plates. Mechanics of Composite Materials and Structures.

